# A Cardiologist’s Clinical Dilemma: An Incidental Finding of a Potentially High-Risk Anomalous Right Coronary Artery Origin

**DOI:** 10.7759/cureus.61375

**Published:** 2024-05-30

**Authors:** Rahul Sharma, Nadim Jaafar, Armin Arbab-Zadeh, Jaideep Patel

**Affiliations:** 1 Internal Medicine, Greater Baltimore Medical Center, Baltimore, USA; 2 Cardiology, Johns Hopkins University, Baltimore, USA; 3 Cardiology, Johns Hopkins Ciccarone Center for the Prevention of Heart Disease, Baltimore, USA

**Keywords:** computed tomography coronary angiogram (ctca), ascending aorta, interarterial course, coronary artery angiography, anomalous origin of right coronary artery, anomalous coronary arteries

## Abstract

Coronary artery anomalies may place patients at risk for various adverse events. We present a case of a 62-year-old male with a two-year history of intermittent chest pain. A computed tomography coronary angiogram revealed a rare finding of an anomalous right coronary artery (ARCA) originating from the left ascending aorta, with high-risk features. This case highlights the complexities in diagnosing and managing ARCA, underscoring the importance of individualized care and careful consideration of invasive intervention risks versus potential benefits.

## Introduction

Coronary artery anomalies (CAAs) are a wide spectrum of congenital abnormalities present in approximately 1 in 100 people. Their clinical implication varies from asymptomatic to more serious complications, including angina, arrhythmias, syncope, or sudden cardiac death (SCD), making their detection critical and warranting further functional testing. Prior research of CAA variants has identified characteristics that increase the risk of complications [1,2]. A continuous challenge faced by cardiologists is the presence of a CAA variant with high-risk features in an asymptomatic individual. Management is often patient-centered, focusing on reducing the risk of complications with medical versus invasive management [3]. We present a scarcely reported case of an anomalous right coronary artery (ARCA) with an abnormal origin and high-risk features, highlighting our approach and challenges in management.

## Case presentation

A 62-year-old male presented to the emergency department with a two-year history of intermittent, non-radiating, retrosternal, self-limiting chest pressure, often postprandial. Given the acceleration and persistence of chest discomfort, now with activity, he sought urgent care. Vitals included a blood pressure of 156/96 mmHg, a heart rate of 77 beats/minute, and oxygen saturation of 99% on room air. Moreover, he was afebrile.

He had a past medical history of hypertension, human immunodeficiency virus, depression, hiatal hernia, and gastritis. His social history was significant for occasional alcohol consumption. He was a non-smoker and had no recreational drug use.

Differential diagnoses included stable angina, acute coronary syndrome, gastroesophageal reflux disease, gastritis, peptic ulcer disease, and a low suspicion for aortic dissection. Hypertension and age accounted for his HEART score of 2.

**Figure 1:**
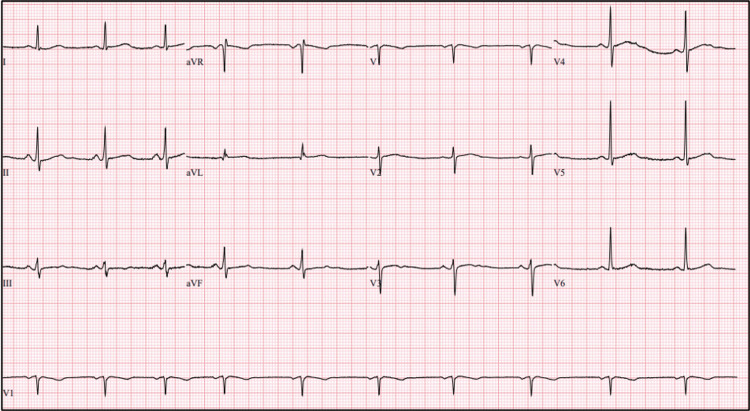
Electrocardiogram revealing a sinus arrhythmia.

**Figure 2:**
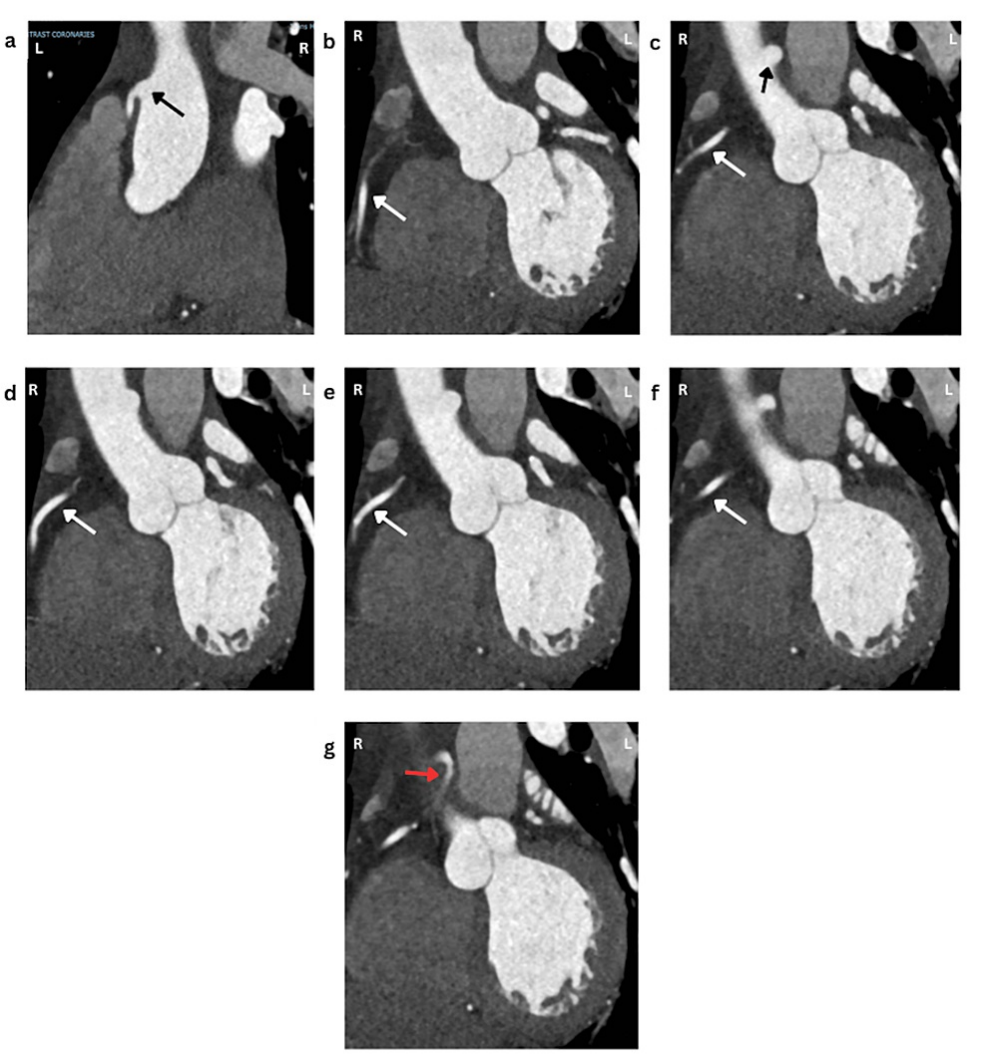
Computed tomography coronary angiogram revealing an anomalous right coronary artery originating superior to the left sinus of Valsalva. (a) The high take-off from the left aortic arch (black arrow). (b-f) The anomalous right coronary artery course (white arrow). (g) A brief interarterial course of the anomalous right coronary artery (red arrow). R: right; L: left

The electrocardiogram revealed normal sinus rhythm with sinus arrhythmia (Figure 1). Cardiac troponin values were normal (normal range: <0.04 ng/mL) and N-terminal pro-B-type natriuretic peptide was normal at 41 pg/mL (normal range: 36-125 pg/mL). Blood work revealed microcytic anemia. Electrolytes and renal function were within normal limits. Outpatient management was suggested. An outpatient computed tomography coronary angiogram (CTCA) revealed an abnormally high origin of the dominant right coronary artery, arising from the left aspect of the ascending aorta, with a short course between the aorta and pulmonary artery, without significant lumen narrowing (Figure 2). His coronary calcium score was 81 with non-obstructive coronary artery atherosclerotic disease involving all three coronary territories, with the most prominent narrowing observed in the proximal ramus. The echocardiogram showed no structural abnormalities with an ejection fraction of 55-60%.

To ensure the absence of inducible myocardial ischemia, he underwent an exercise stress test that was normal. While the presence of an ARCA with the absence of stress-induced ischemic changes does not entirely rule out the anomaly as the cause of chest discomfort, the stress test was reassuring. The patient was managed conservatively with a beta-blocker, given the presence of an interarterial course. He was advised to continue aspirin and atorvastatin for primary prevention of coronary artery disease, given the presence of atherosclerosis. He underwent a gastrointestinal evaluation and was found to have gastritis, esophagitis, and a hiatal hernia, for which he was managed with antacid therapy, yielding improvement of his symptoms. This provided further reassurance that the ARCA was less likely to be responsible for his presenting symptoms.

## Discussion

CAAs are rare congenital defects prevalent in around 1% of the population [1]. Advancements in cardiac imaging, cardiac catheterization, and increased screening have resulted in an increasing number of CAA diagnoses [4]. While often benign, early recognition is important as ARCA can result in life-threatening ischemia, arrhythmias, and SCD. Detection of ARCA, regardless of the clinical context, often warrants an ischemic evaluation [3].

A wide spectrum of CAAs have been recorded and categorized by origin, course, and termination. Our case describes a rarely reported anomalous aortic origin of the coronary artery (AAOCA), accounting for 0.04-0.15% of all CAAs, where the ARCA originates from the left ascending aorta [5].

Imaging plays a key role in characterizing CAA anatomy and risk stratification of AAOCA. Non-invasive anatomic imaging modalities include echocardiography (largely limited to the pediatric population), cardiac MRI, and CTCA, which remains the preferred imaging modality. CTCA offers high spatial resolution, allowing for the accurate visualization of high-risk features, including luminal narrowing, anomalous coronary origin, and the assessment of coronary artery disease, among others [2,6].

The risk of ischemia in CAAs depends on their anatomical features. The milder variants, such as CAA with a retro-aortic or pre-pulmonic course, generally do not require treatment [2]. The high-risk features (see Table 1) have been categorized into fixed and dynamic subtypes. Fixed components include the presence of proximal narrowing or a slit-like ostium, both resulting in reduced perfusion and subsequent ischemia. The dynamic components include the angle of take-off, arterial course (intramural or interarterial), and vessel shape (elliptical or round) [2,7].

**Table 1:** Risk profiles, physiological consequences, and management options in coronary artery anomalies. This is a simplified table that underlines the risk profiles, physiological consequences, and management options in coronary artery anomalies through anatomical features [2,3,7]. *: Exercise restriction or medical management, including beta-blockers or calcium channel blockers, before surgical assessment.

			Management options	
			Symptoms/Positive functional testing	
Risk profile	Anatomical features	Physiological consequence	Yes	No
Low	Pre-pulmonic	Often benign	Conservative management*	Monitor
Low	Retro-aortic	Often benign	Conservative management*	Monitor
Low	High take-off without high-risk features	Often benign	Conservative management*	Monitor
High	Interarterial course	Dynamic compression	Surgical/Interventional assessment	Surgical/Interventional assessment
High	Slit-like ostium	Valve-like occlusion	Surgical/Interventional assessment	Surgical/Interventional assessment
High	Acute take-off angle	Kinking	Surgical/Interventional assessment	Surgical/Interventional assessment
High	Intramural course	Dynamic compression	Surgical/Interventional assessment	Surgical/Interventional assessment
High	Intramural length (long)	Dynamic compression	Surgical/Interventional assessment	Surgical/Interventional assessment
High	Diastolic proximal narrowing/Elliptical vessel shape	Dynamic compression under stress	Surgical/Interventional assessment	Surgical/Interventional assessment
High	Systolic proximal narrowing/Elliptical vessel shape	Dynamic compression under stress and rest	Surgical/Interventional assessment	Surgical/Interventional assessment

In our case, the ARCA originated above the left sinus of Valsalva and had a brief interarterial course (Figure 2). As this variant has scarcely been reported in the literature, its role in SCD has not been established. The interarterial course, until recently, was thought to cause ischemia by compression between the aorta and pulmonary artery. However, this has been disputed, as the aortic and coronary pressures are greater than the pulmonary artery pressure, making compression by the great vessels unlikely. Studies suggest that intramural segments increase the risk of hypoperfusion and ischemia due to compression during systole, which is extenuated during strenuous exercise [2,8]. This, however, does not explain the increased association of SCD in those with an interarterial course and no intramural segment.

Current consensus recommendations on CAAs include a functional/physiologic assessment and evaluation for an alternative etiology in symptomatic patients [2]. Our patient underwent exercise stress testing, which revealed no signs of ischemia and guided a conservative approach with medication.

Overall, treatment is often tailored based on presenting symptoms and anatomical features of the AAOCA. The decision regarding surgical or percutaneous intervention for patients with AAOCA depends largely on the presence of symptoms and evidence of inducible myocardial ischemia. Unfortunately, there are no high-quality, prospective studies that investigated the value of revascularization in the setting of AAOCA. Given that AAOCA has not been found to be conclusively the cause of SCD in patients over the age of 30, some advocate reserving revascularization for younger patients. Many still favor revascularization in patients with AAOCA and associated angina, syncope, ventricular arrhythmias, history of cardiac arrest, or positive stress imaging but the supporting evidence is limited​ [2,3].

Revascularization poses the risks of invasive procedures and the dilemma for cardiologists remains in weighing those risks against potential but unproven benefits. A conservative approach involving the use of beta-blockers appeared reasonable in the presented case of a 62-year-old man without inducible myocardial ischemia.

## Conclusions

We present a case of AAOCA with an interarterial course. Given the lack of prospective studies, the most appropriate management remains unclear. The risks of revascularization should be carefully weighed against assumed benefits, with a multidisciplinary approach promoting individualized patient care. We highlight an existing gap in the literature that requires further prospective studies to address medical versus surgical management in this clinical dilemma.
